# Assessment of venous thromboembolism in adult-type diffuse gliomas at a quaternary neuro-oncology center: a retrospective cross-sectional study and systematic review

**DOI:** 10.1007/s10143-026-04299-6

**Published:** 2026-04-25

**Authors:** Leonardo de Sousa Bernardes, Lucas de Oliveira Woehl, Jean Gonçalves de Oliveira, José Carlos Esteves Veiga, João Luiz Vitorino Araujo

**Affiliations:** 1 Department of Neurology, Santa Casa de São Paulo School of Medical Sciences, São Paulo, SP Brazil; 2 Universidade do Planalto Catarinense, Lages, SC Brazil; 3 Division of Neurosurgery, Deparment of Surgery, Irmandade da Santa Casa de Misericórdia de São Paulo (ISCMSP) and Santa Casa de São Paulo School of Medical Sciences (FCMSCSP), São Paulo, SP Brazil; 4 Hospital do Coração (HCOR), São Paulo, SP Brazil

**Keywords:** GliomaFF, Glioblastoma, Astrocytoma, Oligodendroglioma, Venous Thromboembolism, Central Nervous System Neoplasms

## Abstract

Venous thromboembolism (VTE) remains a clinically relevant and impactful complication in patients with adult-type diffuse gliomas, and its overall frequency and determinants remain poorly defined. We retrospectively examined 147 patients with gliomas treated at a Brazilian quaternary neuro-oncology center during 2018–2023 and performed a Preferred Reporting Items for Systematic Reviews and Meta-Analysis/Synthesis without Meta-Analysis–based systematic review of observational studies (incorporating eight cohorts; *N* = 7779) published since 2015. The patients’ median age at diagnosis was 55 (range 20–86) years. Glioblastoma was the predominant glioma type (*n* = 91, 61.9%), followed by astrocytoma (*n* = 32, 21.8%) and oligodendroglioma (*n* = 24, 16.3%). VTE events occurred in 5 (3.4%) patients, 4 (80%) with isocitrate dehydrogenase (IDH)–wild-type glioblastoma (5.6% incidence within this subgroup) and 1 (20%) with IDH-mutant astrocytoma; no thrombosis was identified in patients with oligodendrogliomas. All five events (three pulmonary thromboembolism [PTE] and two lower-limb deep-vein thrombosis [DVT]) events) occurred within 6 months of surgery, clustered in patients aged between 41 and 60 years and evenly distributed by sex. Cerebral venous thrombosis (CVT) was not observed. In the systematic review, the VTE incidence rate was 6.2%–31% in grade 4 gliomas and 1.4%–5.2% in grades 2 and 3. DVT comprised 60% of thromboembolic events; PTE, 35%; and CVT, 5%. Poor performance status, a surgery duration of > 4 h, and bevacizumab exposure increased the risk for VTE, whereas IDH mutation reduced it by ~ 70%. The median time from surgery to VTE diagnosis was 21–90 days, and 30-day mortality after VTE was 7%–15%. Findings show that large, prospective, multicenter, molecularly annotated glioma cohorts remain vital for thrombosis risk stratification and targeted prophylactic strategy development.

Clinical trial number: Not applicable.

**Supplementary Information:**

The online version contains supplementary material available at 10.1007/s10143-026-04299-6.

## Introduction

In 2021, the World Health Organization (WHO) released the fifth edition of the classification of central nervous system (CNS) tumors, aiming to standardize their nomenclature and incorporate molecular discoveries since the 2016 edition [1]. Gliomas represent approximately 80%–85% of malignant primary CNS tumors, with glioblastomas accounting for about 49% of malignant brain tumors, while diffusely infiltrating lower-grade gliomas comprise approximately 30% of cases [2]. Based on WHO guidelines, adult-type diffuse gliomas are classified as grades 2–4 based on their biological aggressiveness, with IDH-wild-type glioblastomas being defined as grade 4 and IDH-mutated astrocytomas and oligodendrogliomas as grades 2–4 and as grades 2 and 3, respectively [3].

**Fig. 1:**
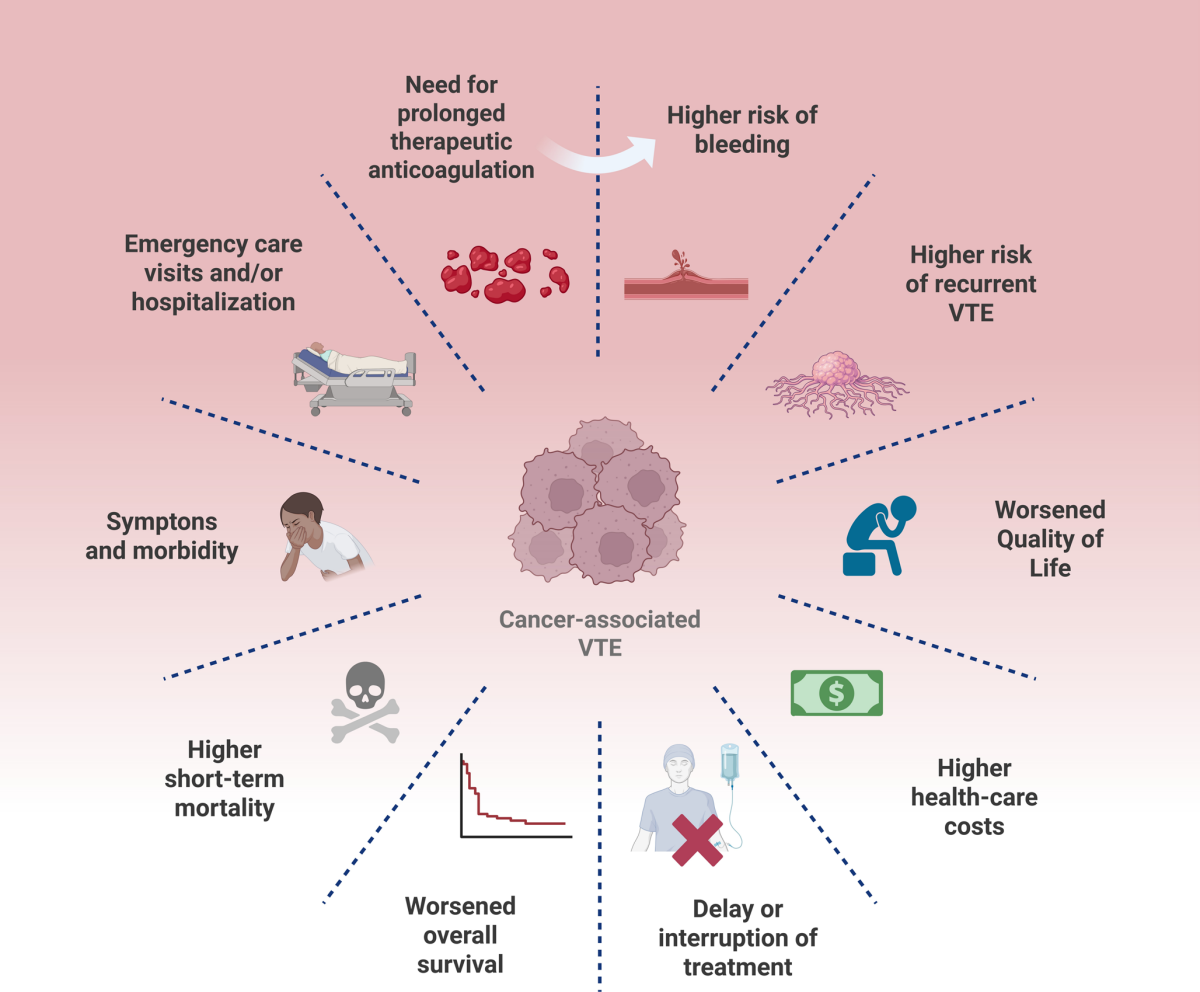
Main consequences of cancer-associated VTE. Cancer-associated VTE is linked to worse outcomes and poor prognosis, such as higher morbidities, the risk of bleeding, the recurrence of VTE, and higher healthcare costs. Picture created on biorender.com. VTE: venous thromboembolism

The risk factors of gliomas are limited, with age and prior exposure to ionizing radiation (particularly in childhood) being the most established ones [4]. Allergic and atopic conditions may be inversely associated, and cell phone radiation or occupational exposures are not considered significant [4]. Thromboembolic events affect up to 60% of patients with brain neoplasms [5, 6], and approximately 30% of patients with gliomas have deep-vein thrombosis (DVT) or pulmonary thromboembolism (PTE), especially within 6 months of diagnosis [7, 8]. These events increase mortality by 30% within 2 years [9], as shown in Fig. 1, and brain tumors have one of the highest thromboembolic rates among malignancies, second only to pancreatic cancer [10, 11].

In a Canadian study [10], 7.4% of 163 patients with glioblastoma developed cerebral venous thrombosis (CVT), an underreported but serious complication. Watanabe et al. [12] reported venous thromboembolism (VTE) in 26.7% of patients with gliomas, mostly asymptomatic and detected through elevated D-dimer levels. The risk factors of VTE include age > 75 years; immobility; comorbidities; prior thrombosis [2]; blood groups A, B, and AB; and high levels of Von Willebrand Factor and factor VIII, although data remain conflicting [2, 13]. Patients with high-grade gliomas, such as glioblastoma, known for elevated prothrombotic mediators (e.g., tissue factor [TF], podoplanin), are especially prone to VTE [13]. A tumor size of > 5 cm, subtotal resection, intraluminal thrombosis, and recurrence further contribute to risk [2].

However, VTE remains a clinically relevant and impactful complication in adult-type diffuse gliomas, and to date, no investigation has included or characterized a Latin American cohort, resulting in a lack of regional data to inform clinical awareness and management in this population. This study aimed to assess the frequency of thromboembolic events in patients with adult-type diffuse gliomas treated at a quaternary neuro-oncology center in Brazil. We identified patterns related to histological subtypes, age groups, and sex. We also conducted a systematic review of observational studies to provide a robust qualitative overview of the available data.

## Materials and methods

### Retrospective cross-sectional study in a quaternary neuro-oncology center

#### Study design and population

This was a descriptive, retrospective, cross-sectional study carried out in a reference center, with patients treated in the neuro-oncology outpatient clinic of the neurosurgery discipline of the Irmandade da Santa Casa de Misericórdia de São Paulo from January 2018 to December 2023.

#### Inclusion and exclusion criteria

Patients were included if they were over 16 years old; diagnosed with adult-type diffuse glioma (glioblastoma, astrocytoma, or oligodendroglioma) confirmed through anatomopathological study, according to the WHO CNS 2021 classification; and treated in the neuro-oncology outpatient clinic. Patients under 16 years of age were excluded. Only symptomatic patients (those who presented with tachycardia, dyspnea, chest pain, decreased oxygenation, and/or lower-limb edema) were investigated for VTE events, which were diagnosed based on imaging findings (venous Doppler ultrasonography for DVT and computed tomography [CT] pulmonary angiography for PTE).

#### Sources and data collection

Patient data were collected from the institution’s electronic medical record (MV system). Data pertinent to each patient were anonymously cataloged using a code for each patient. The variables collected regarding the study participants were sex, type of neoplasia (as determined through anatomopathological and immunohistochemical studies), and the presence of a thromboembolic event, such as DVT, PTE, or CVT.

#### Statistical analysis

For categorical variables, the chi-square and Fisher’s exact tests were performed. Both tests evaluate whether the variables involved can be considered independent of each other (hypothesis [H]0: variables are independent × H1: variables are related). The chi-square test follows the premise that all cells resulting from crossing the categories have more than five cases under variable independence (expected cells). In this study, when this premise was not met, we conducted Fisher’s exact test. Just like in analysis of variance (ANOVA), the test response does not indicate in which groups there is or is not a difference. When the test statistic was significant (*p* < 0.05), the multiple comparison test with Bonferroni adjustment was performed. For all cases, a significance level of 5% was considered, that is, the null hypothesis (H0) was rejected when *p* < 0.05 (the result was statistically significant at a 5% significance level). To evaluate the difference in sample characteristics according to VTE, the *t*-test was performed for age and the chi-square or Fisher’s exact test for categorical variables. The *t*-test has similar characteristics as the aforementioned ANOVA but is used when there are two groups to be compared. IBM SPSS version 20 software was used for statistical analysis.

### Systematic literature review

#### Study selection

**Fig. 2:**
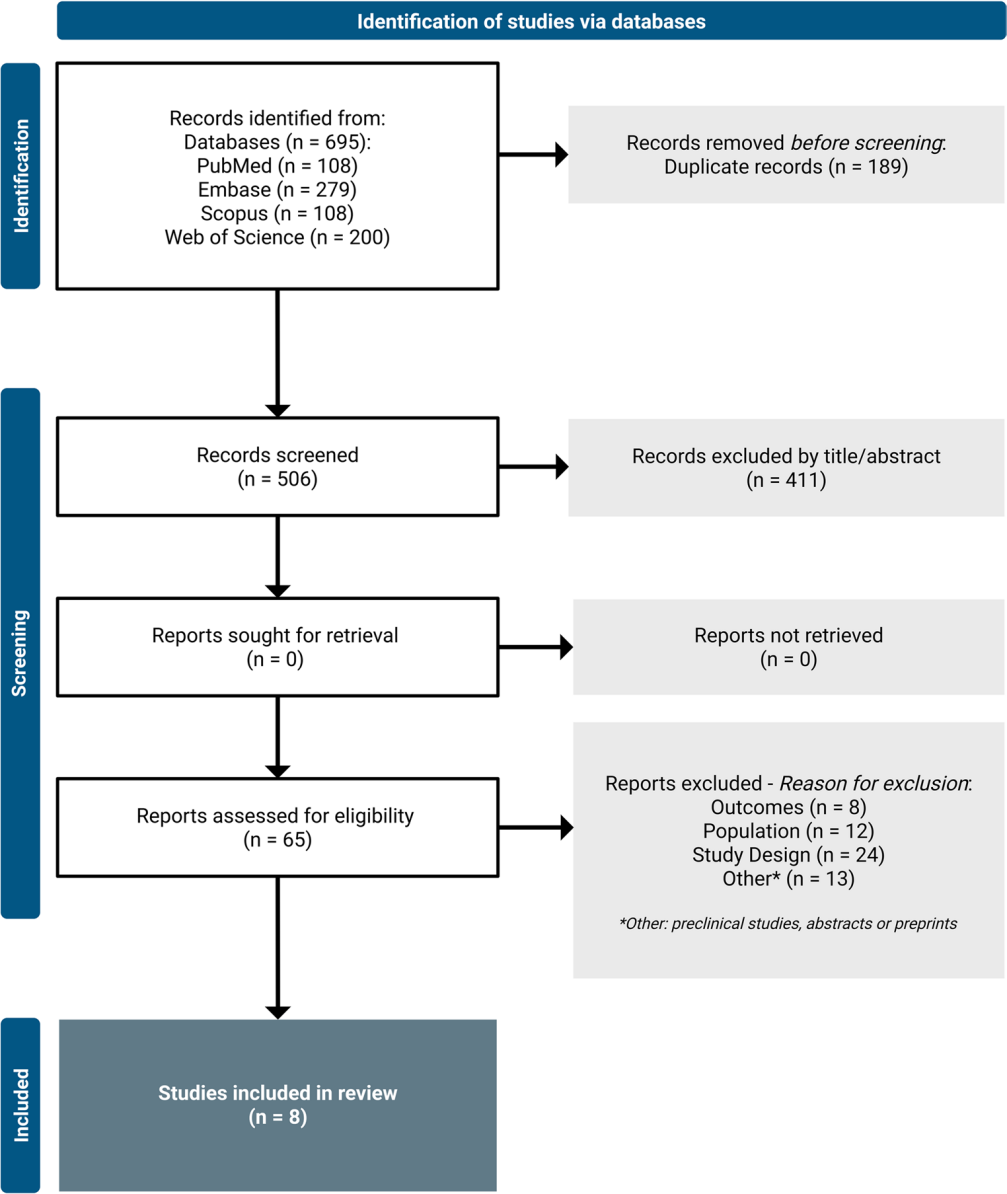
PRISMA flow diagram showing the selection and inclusion of studies in the systematic review. PRISMA: Preferred Reporting Items for Systematic Reviews and Meta-Analyses

This systematic review was conducted in accordance with the Preferred Reporting Items for Systematic Reviews and Meta-Analysis (PRISMA) and the Synthesis without Meta-Analysis (SWiM) reporting guidelines (Fig. 2) [14, 15]. Studies were identified by searching the PubMed, Embase, Scopus, and Web of Science from inception to November 2024. The search was structured around the main concepts (gliomas and VTE) using associated Health Science Descriptors/Medical Subject Headings (DeCS/MeSH), combined descriptors, entry terms, and free vocabulary, arranged with the Boolean operators AND and OR, as shown in Online Resource 1.

#### Eligibility criteria

The inclusion criteria were as follows:

- Studies with full text available and published in English, Spanish, or Portuguese.
- Observational, cross-sectional, prospective, and retrospective studies.
- Studies reporting outcomes of interest.
- Studies on VTE in patients with adult-type diffuse gliomas.

The exclusion criteria were as follows:

- Case reports.
- Preclinical studies.
- Preprints of any type of study.
- Studies without a report on any of the predefined data of interest.

The results were limited from 2015 to November 2024 to capture the most recent decade of evidence and ensure that the data reflect current trends, as older studies may be outdated due to changes in technology, diagnostic criteria, and glioma classification.

#### Outcomes of interest

Incidence- and prevalence-related outcomes were analyzed separately, with incidence defined as the rate of new VTE events over time and prevalence representing the cumulative proportion of patients diagnosed with VTE at any point during their disease course. This distinction allowed for a clearer interpretation of thrombotic risk across studies. Risk factors associated with VTE in adult-type diffuse gliomas were also assessed, including clinical variables, such as age, performance status, and surgical interventions; biological markers, such as coagulation factors and molecular tumor characteristics; and pharmacologic effects.

#### Study triage and data extraction

Two researchers independently screened articles for inclusion criteria and extracted data from full texts and published appendices of the included studies. Each researcher independently checked the accuracy of the other’s data extraction. Any disagreements were resolved through consensus or, if necessary, by a third author. The authors extracted baseline characteristics of the studies and the prespecified outcomes. We also extracted baseline characteristics of patients, such as age, gender, demographic data, tumor subtype, and glioma WHO grade, along with the mean follow-up duration and patient age range.

#### Data synthesis

We performed a qualitative synthesis of the included studies, focusing on incidence rates, prevalence estimates, and risk factors of VTE in patients with adult-type diffuse gliomas. To ensure methodological rigor, incidence and prevalence data were reported separately to distinguish between the rate of new thromboembolic events over time and the overall burden of VTE in this patient population. For categorical data, we extracted and compared frequency distributions and proportions across studies. We also reported risk estimates, including odds ratios (ORs) and hazard ratios (HRs), along with their 95% confidence intervals (CIs), when available. To enhance accuracy, we considered *p*-values, with statistical significance set at *p* < 0.05. Given the variability in study designs, patient populations, and follow-up durations, a formal quantitative synthesis (meta-analysis) was not performed. Instead, we critically assessed and stratified the included studies into categories of incidence, prevalence, and risk factors in order to improve interpretability and clinical relevance.

#### Quality assessment and risk of bias

Two independent authors conducted an assessment of the methodological quality and risk of bias of the included studies using the Newcastle–Ottawa Scale (NOS) [16]. The NOS assesses the design quality of nonrandomized studies, including case–control and cohort studies. Scores were assigned for selection criteria, comparability, and outcome (cohort) or exposure (case–control), with an overall score out of 9. The overall risk of bias in a study was considered high, of some concern, or low, depending on the score. Studies were considered to have a high overall risk of bias if one domain (selection criteria, comparability, or outcome) received a high risk-of-bias score.

#### Human and animal rights

This study complied with ethical standards according to the Declaration of Helsinki (as revised in 2024) and relevant national regulations.

## Results

### Results of the retrospective cross-sectional study

The sample consisted of 147 patients, comprising 83 (56.5%) men and 64 (43.5%) women, aged between 20 and 86 years. The majority of patients (*n* = 59, 40.1%) were 41–60 years old at the time of diagnosis, while 58 (39.5%) were ≥ 61 years old, and 30 (20.4%) belonged to the youngest age group (20–40 years). The patients’ sociodemographic profile is summarized in Table 1.

**Table 1:** Sociodemographic characteristics of the 147 study participants

Characteristics		Glioblastoma	Astrocytoma	Oligodendroglioma
n		91	32	24
Gender	Male	55 (60%)	13 (49%)	15 (63%)
	Female	36 (40%)	19 (59%)	9 (38%)
Age (mean ± SD)		60.7 ± 11.9	38.9 ± 12.6	47.6 ± 13.6
Age Group (years)	20–40	5 (5%)	19 (59%)	6 (25%)
	41–60	34 (37%)	11 (34%)	14 (58%)
	≥ 61	52 (57%)	2 (6%)	4 (17%)
VTE	No	87 (96%)	31 (97%)	24 (100%)
	Yes	4 (4%)	1 (3%)	0 (0%)

*n* number of patients, *SD* standard deviation, *VTE *venous thromboembolism

**Fig. 3:**
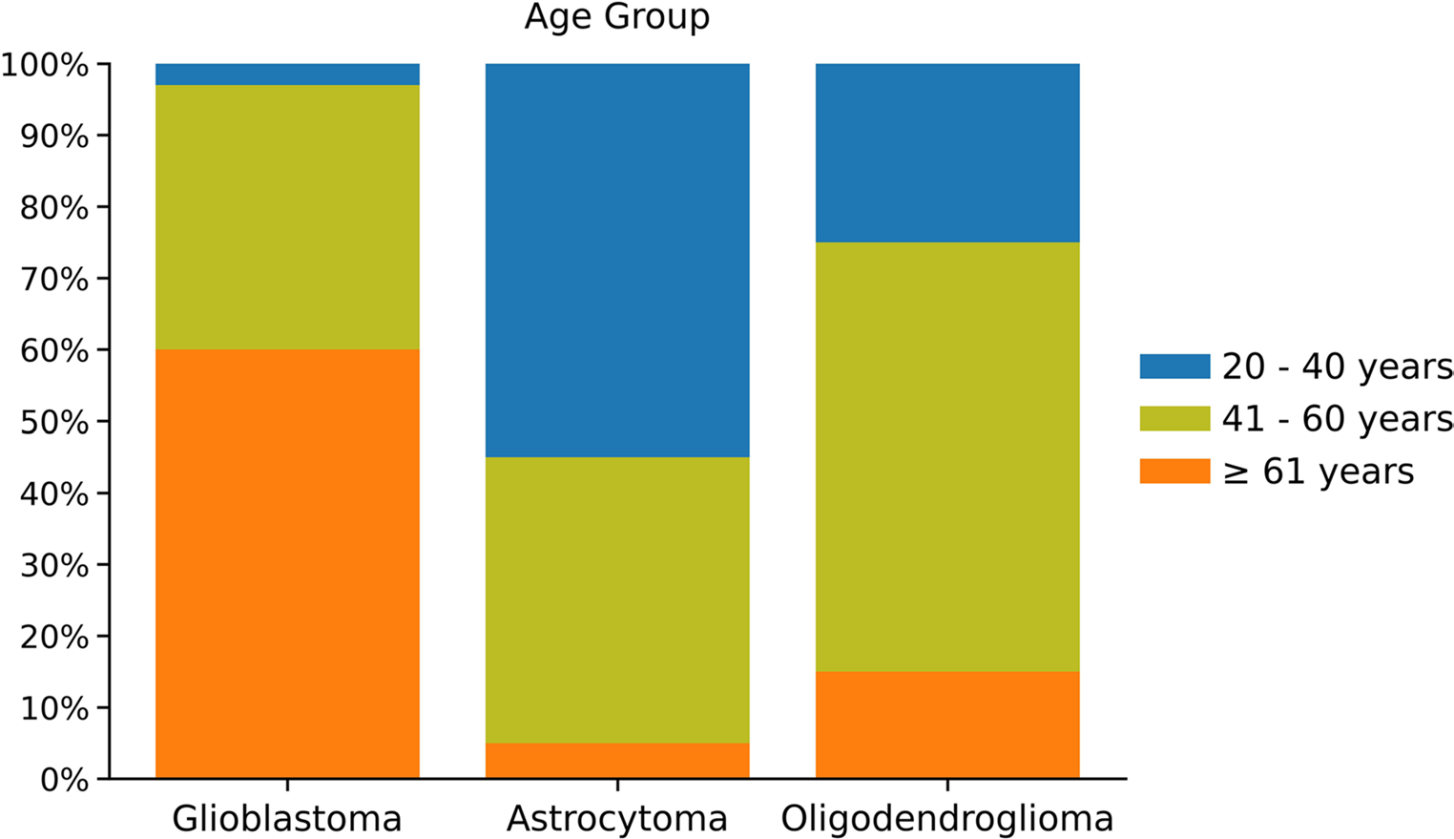
Prevalence of adult-type diffuse gliomas in the study patients by age group

Histological subtype analysis results showed that glioblastoma was the most frequently found adult-type diffuse glioma (*n* = 91, 61.9%), followed by astrocytoma (*n* = 32, 21.8%) and oligodendroglioma (*n* = 24, 16.3%). Stratification by patient age showed that in the age group of 20–40 years, adult-type diffuse astrocytoma was the most common histological type of neoplasm, followed by oligodendroglioma and IDH-wild-type glioblastoma. However, in older adults (≥ 61 years old), IDH-wild-type glioblastoma was, by far, the most recurrent type of neoplasm compared to oligodendroglioma and astrocytoma, as illustrated in Fig. 3.

Thromboembolic events were found in 5 (3.4%) of the 147 patients, 4 (80%) of whom had IDH-wild-type glioblastoma (5.6% incidence within this subgroup) and 1 (20%) had IDH-mutated astrocytoma (Table 2). All patients diagnosed with VTE received therapeutic anticoagulation with unfractionated heparin followed by warfarin. No life-threatening bleeding or anticoagulation-related complications were observed during treatment. Because only five VTE events were observed, the study was underpowered to detect statistically significant associations between VTE and demographic or tumor-related variables. Thus, the statistical result (test) was used to interpret VTE for the population (there was no proof of a statistically significant difference); however, what was observed could be described as a specific result for this sample. Furthermore, 3 (75%) of the 4 patients diagnosed with glioblastoma presented with PTE, while the fourth patient, and the only representative of the IDH-mutated adult-type diffuse astrocytoma group, presented with DVT. All VTE events occurred within 6 months of surgery.

**Table 2:** Statistical analysis of VTE prevalence among participants. VTE: venous thromboembolism

Characteristics		No	Yes	*p*-value	Tests
n		142	5	-	-
Gender	Male	81 (57%)	2 (40%)	0.653	Fisher’s exact
	Female	61 (43%)	3 (60%)		
Age (mean ± SD)		54 ± 15.4	49 ± 12.9	0.478	t-test
Age Group (years)	20–40	29 (20%)	1 (20%)	0.841	Fisher’s exact
	41–60	56 (39%)	3 (60%)		
	≥ 61	57 (40%)	1 (20%)		
Neoplasia	Glioblastoma	87 (61%)	4 (80%)	0.825	Fisher’s exact
	Astrocytoma	31 (22%)	1 (20%)		
	Oligodendroglioma	24 (17%)	0 (0%)		

*n* number of patients, *SD* standard deviation

Of the 5 (3.4%) patients who were diagnosed with VTE, 3 (60%) were women, whose ages were 28, 56, and 62 years, respectively, while the 2 (40%) male patients were 48 and 52 years old, respectively.

### Results of the systematic review

A total of 695 potentially relevant citations were identified. Rayyan software was used to detect potential duplicates, and the reviewers resolved them manually, verifying and excluding them one by one. Following the removal of 189 (27.2%) duplicates, 506 (72.8%) records were screened, of which 411 (81.2%) were excluded based on their titles and abstracts. A total of 65 full texts were assessed for their eligibility. The main reason for exclusion was study design; the reason for the exclusion of each study is shown in Online Resource 2. 

Eight (12.3%) studies [10, 17–23] met the criteria for eligibility and were incorporated into the review (Fig. 2). The majority were retrospective cohort studies (*n* = 7, 87.5%), with only 1 (12.5%) prospective cohort study [23]. They were published in North America (*n* = 5, 62.5%), Europe (*n* = 2, 25%), and Asia (*n* = 1, 12.5%).

#### Participants

A total of 7779 patients were included across the 8 (12.3%) studies. Sample sizes varied widely, ranging from small cohorts of 107 (1.4%) individuals [23] to large multicenter studies [19] involving 3630 (11.7%) patients. The participants’ weighted mean age across all studies was 54.05 ± 3.85 years. The studies spanned different periods, ranging from 2000 to 2021. The male-to-female ratio varied, from nearly equal distribution in some cohorts to a more pronounced male predominance in others, as observed in the dataset from Denmark [19]. VTE definitions and ascertainment methods varied across studies but were generally based on imaging-confirmed events (e.g., Doppler ultrasonography, CT pulmonary angiography, or MRI). Four studies identified VTE events through clinical records [18], retrospective chart review [20], national registry databases [19], or objectively documented outcomes without explicitly reported imaging modalities [23]. No study reported systematic screening for asymptomatic VTE. Table 3 summarizes the baseline characteristics and key findings of the included studies.

**Table 3:** Baseline characteristics and key findings of the included studies

Author	Year	Region	Study design	Sample size, *n*	Sex (M/F)	Mean age, years	Study period	VTE definition and diagnostic method	Outcome measured	Key findings
Diaz et al.	2021	United States	R	635	325 / 265	54.58	2005–2017	Radiologically confirmed DVT, PE, or CVST	Incidence of VTE, molecular risk factors (IDH, MGMT), and survival	VTE incidence ranging from 15.7% to 42.2%, higher in glioblastoma; absence of IDH mutation associated with increased risk; MGMT status did not influence risk
Eisele et al.	2022	Switzerland	R	414	261 / 153	NA	2005–2014	VTE identified from clinical records^a^	Incidence of VTE, risk factors, treatment complications, and survival	VTE in 15.7% of patients; occurs early (median 1.8 months after diagnosis); no impact on survival; hemorrhagic complications associated with anticoagulation
Helmi et al.	2019	Canada	R	163	107 / 56	53.82	2009–2015	DVST detected on MRI	Incidence of CVT, radiological risk factors, and survival	CVT in 7.4% of patients, often before treatment; involvement of venous sinuses increases risk; no significant impact on survival
Hovman et al.	2024	Denmark	R	3,630	1,848 / 1,235	NA	2010–2018	DVT or PE identified through national registry records^b^	Incidence of VTE, risk factors, and survival	VTE incidence of 5.2%–6.8%; higher risk in the first 3 months; advanced age, male sex, and poor functional status increase risk
Mandel et al.	2021	United States	R	282	176 / 106	54	2000–2013	VTE identified through retrospective chart review^c^	Incidence of VTE, molecular factors (IDH), and time to event	VTE incidence of 18.4%; IDH mutation was not a statistically significant factor; trend toward higher risk in IDH wild-type tumors
Nabi et al.	2016	United States	R	1002	590 / 412	57.2	2001–2011	Symptomatic DVT (venous duplex) or PE (CT angiography/V/Q scan)	Incidence of VTE, clinical risk factors, and functional impact	VTE in approximately 24% of patients; higher risk associated with advanced age, multiple hospitalizations, poor performance status (PS), and bevacizumab use
Shi et al.	2021	China	R	492	254/238	46.42	2018–2021	Symptomatic DVT confirmed by Doppler USG	Incidence of DVT, perioperative risk factors, and clinical outcomes	DVT incidence of 14.8%; preoperative factors (age, D-dimer, surgical time) increased risk; prophylactic anticoagulation did not increase bleeding
Streiff et al.	2015	United States	P	107	55 / 52	57	2005–2008	Objectively confirmed symptomatic VTE^d^	Incidence of VTE, clinical and laboratory risk factors, and time to event	VTE incidence of ~ 24%; higher risk in the first 6 months; elevated factor VIII and tumor biopsy associated with increased risk

*CT  *Computed Tomography, *n *number of patients, *M *male,* F* female,* NA* not available, *DVT *Deep vein thrombosis, *DVST *Dural venous sinus thrombosis, *IDH* Isocitrate dehydrogenase, *PE*  Pulmonary embolism, *P* Prospective, *R* Retrospective, *USG* ultrasonography,*VTE *Venous thromboembolism, *WHO *World Health Organization

^a^ VTE events were identified retrospectively from clinical records in a population-based cohort; specific diagnostic imaging modalities were not detailed

^b^ VTE diagnoses were obtained from the Danish National Patient Registry using validated diagnostic codes for DVT and PE

^c^ VTE events were identified through retrospective chart review of astrocytoma patients enrolled in the PROACTIVE cohort; specific imaging modalities were not explicitly described

^d^ The study outcome was defined as objectively documented symptomatic VTE in a prospective cohort; the specific diagnostic imaging modality used for confirmation was not detailed in the methods

#### Incidence and prevalence of VTE

Several studies have examined the incidence rates of VTE in patients with gliomas. Diaz et al. [17] found that the cumulative incidence of VTE over a median follow-up of 17.9 months was 8.2% (95% CI 5.4–11.1) in WHO grade 2, 9.2% (95% CI 6.1–12.5) in grade 3, and 30.8% (95% CI 27.1–34.6) in grade 4 gliomas. Hovman et al. [19] reported a VTE incidence of 5.2% (95% CI 4.1–14.8) in WHO grade 2, 6.3% (95% CI 5.1–12.3) in grade 3, and 6.8% (95% CI 6.1–7.9) in grade 4 gliomas, with the highest risk occurring within the first 3 months after diagnosis. Shi et al. [22] specifically analyzed postoperative thrombotic complications, showing that 14.84% (95% CI 12.3–17.9) of patients with gliomas developed DVT after craniotomy. Streiff et al. [23] estimated the hazard rate of VTE in patients with newly diagnosed high-grade gliomas at 0.15 per person-year, with 24% (95% CI 17–34) of the patients developing symptomatic VTE during a median survival of 17.7 months. Eisele et al. [18] found that 15.7% (95% CI 12.6–18.9) of patients with glioblastoma were diagnosed with VTE, with a significant proportion of them developing VTE within 35 days postoperatively, indicating the heightened risk during the perioperative period. Mandel et al. [20] reported VTE occurring in 18.4% (95% CI 14.1–22.6) of patients with astrocytomas, with no statistically significant difference between IDH-mutant and IDH-wild-type tumors (*p* = 0.41). Nabi et al. [21] reported that 16.2% (95% CI 13.1–19.5) of patients with glioblastoma experienced VTE. Table 4 shows a summary of VTE, DVT, and PTE in patients with adult-type diffuse gliomas across the studies included in our systematic review.

**Table 4:** Summary of VTE, DVT, and PTE in patients with adult-type diffuse gliomas across the included studies. DVT: deep-vein thrombosis; PTE: pulmonary thromboembolism; VTE: venous thromboembolism

Author	Year	*n*	VTE	DVT	PE	Other
Hovman et al.	2024	3,630 WHO Grade II: 230 WHO Grade III: 317 WHO Grade IV: 3,083	314 (8.65%) WHO Grade II: 12 (5.2%) WHO Grade III: 20 (6.3%) WHO Grade IV: 208 (6.8%)	WHO Grade II: 43% WHO Grade III: 58% WHO Grade IV: 35%	Grade II: 57% Grade III: 42% Grade IV: 65%	NA
Mandel et al.	2021	282 Astrocytoma WHO Grade II: 11 (3.9%) Astrocytoma WHO Grade III: 14 (5%) GBM (WHO Grade IV): 249 (88.3%)	52 (18.4%)	26 (50%)	10 (19.2%)	16 (30.8%)
|Eisle	2022	414 *GBM only (WHO Grade IV)*	65 (15.7%)	26 (40.6%)	29 (45.3%)	DVT + PE = 7 (10.9%)
Shi et al.	2021	492	76 (15.4%)	73 (96%)	3 (1.2%)	DVT + PE = 3 (0.6%)
Diaz et al.	2021	635 WHO Grade II: 147 WHO Grade III: 109 GBM (WHO Grade IV): 334	125 (19.6%) WHO Grade II: 12 (8.2%) WHO Grade III: 10 (9.2%) WHO Grade IV: 103 (30.8%)	NA	NA	NA
Helmi et al.	2019	163* *GBM only (WHO Grade IV)*	12 (7.4%)	NA	NA	CVT = 12 (7.4%)
Nabi et al.	2016	1002* *GBM only (WHO Grade IV)*	162 (16.1%)	61 (38%)	91 (56%)	DVT + PE = 10 (6%)
Streiff et al.	2015	107 GBM: 91 (WHO Grade IV) WHO Grade III: 14 Glioma NOS: 2	26 (24.2%) GBM (WHO Grade IV): 23 (26%)	25	1	DVT + PE = 0

*n* number of patients, *NA* not available, *GBM* glioblastoma multiforme, *PE* pulmonary embolism, *VTE* venous thromboembolism, *CVT* cerebral venous thrombosis, *DVT* deep vein thrombosis, *WHO* World Health Organization, *NOS* Not otherwise specified

#### Risk factors of VTE

Multiple studies have identified independent risk factors associated with an increased likelihood of VTE in patients with gliomas. In this review, age was consistently highlighted as a major risk factor, with patients aged 56–65 years exhibiting an OR of 7.86 (95% CI 3.63–17.03, *p* < 0.001) for developing DVT, as shown by Shi et al. [22]. Postoperative factors were also found to be critical predictors. A longer surgery duration was associated with a significantly increased risk for DVT (OR 2.87, 95% CI 1.6–5.07, *p* < 0.001), reinforcing the need for early mobilization and prophylaxis in neurosurgical patients [18]. Biological factors also contributed to thrombotic risk. Streiff et al. [23] found that patients with an initial tumor biopsy had a 3-fold increased risk of developing VTE (HR 3.0, 95% CI 1.2–8.8, *p* = 0.02), while elevated factor VIII levels were associated with a 2.1-fold increased likelihood of thrombosis. Regarding pharmacologic influences, Nabi et al. [21] demonstrated that bevacizumab use increased the risk for VTE by 1.79 times (95% CI 1.21–2.64, *p* < 0.001). These findings highlight the complex balance between anti-angiogenic therapy and thrombotic risk, requiring careful risk–benefit assessment when prescribing such agents.

#### Risk of bias across studies

**Fig. 4:**
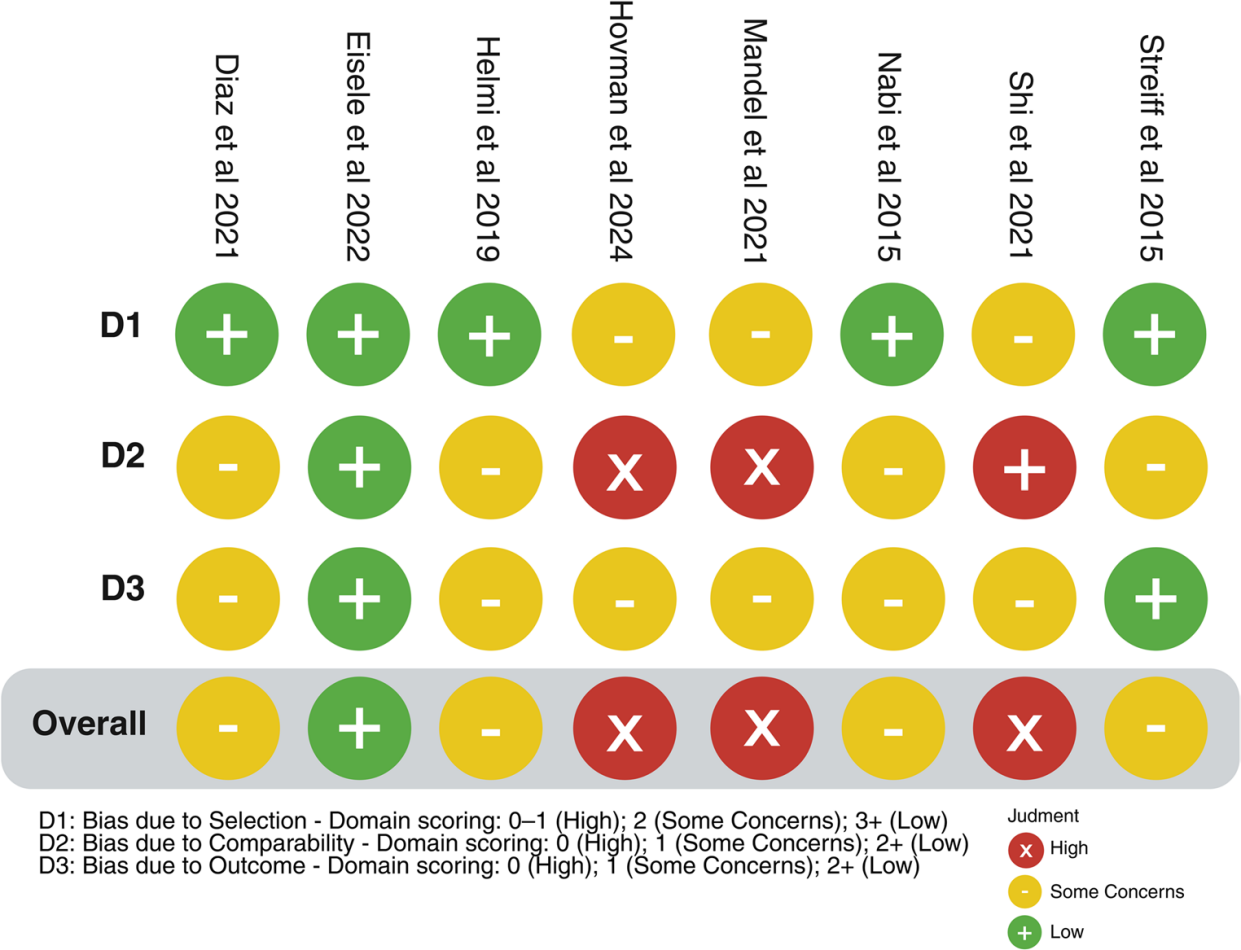
Risk-of-bias assessment for each study according to NOS scores. NOS: Newcastle–Ottawa Scale

Based on the NOS scores, of the 8 (12.3%) included studies, 3 (37.5%) [19, 20, 22] were classified as having a high risk of bias, primarily due to issues in comparability and outcome assessment (Fig. 4), while 4 (50%) [10, 17, 21, 23] were rated as having a moderate risk of bias, largely due to selection bias and comparability concerns. The remaining study [18] was assessed as having a low risk of bias.

## Discussion

Although the association between VTE and adult-type diffuse gliomas is well recognized in the literature, we were able to identify only eight studies [10, 17–23] whose primary objective was to assess the prevalence of VTE and determine the most commonly associated risk factors—an approach similar to ours. To the best of our knowledge, this study represents the first Latin American cohort investigation on VTE in adult diffuse-type gliomas, as well as the first to complement institutional data with a structured systematic review of the literature.

### Incidence and prevalence of VTE

A large retrospective Danish cohort study [19] involving 3630 patients with adult-type diffuse gliomas, grades 2–4, reported that, histologically, the majority of patients had grade 4 gliomas (84.9%), who also showed the highest rate of thromboembolic events (6.8%) compared to patients with grade 3 (6.3%) and grade 2 (5.2%) gliomas. In a single-center retrospective cohort study, Diaz et al. [17] evaluated the frequency of VTE in 635 patients, including 301 (47.4%) with lower-grade gliomas and 334 (52.6%) with IDH-wild-type glioblastomas. Notably, the presence of IDH mutation was associated with a 3-fold lower prevalence of VTE compared to patients without the mutation. In addition, patients with grade 4 adult-type diffuse gliomas exhibited a higher rate of hemiparesis (19.5%) compared to those with grade 3 (13.8%) and grade 2 (6.1%) gliomas. Furthermore, 27 (21.6%) VTE events occurred within the first 30 days following surgical intervention. Our study also observed a predominance of thromboembolic events among patients with IDH-wild-type glioblastomas, reinforcing the association already suggested in the literature between the absence of the IDH mutation and increased thrombotic risk. Furthermore, although we did not systematically assess neurological deficits, such as hemiparesis, the predominance of grade 4 gliomas among the VTE cases in our cohort may indirectly reflect a correlation between higher tumor aggressiveness, functional impairment, and elevated thrombotic risk. Despite methodological and sample size differences across studies, the findings consistently highlight that both the molecular profile and the histological grade of the tumor are important factors in assessing VTE risk in patients with adult-type diffuse gliomas.

Another single-center retrospective cohort study [18] involving 414 patients diagnosed with glioblastoma and a median follow-up of 10.8 months found that thromboembolic events occurred in 65 (15.7%) patients, with a median time to diagnosis of 1.8 months. Among the 65 patients with documented VTE, 26 (40.6%) had DVT, 29 (45.3%) had PTE, 7 (10.9%) had both, and 2 (3.1%) presented with CVT; in 1 (1.5%) patient, the specific site of thrombosis was not identified. In our sample, the prevalence of VTE was 3.4%, which may be explained, at least in part, by the small cohort size, the heterogeneity of the included histological subtypes, and the absence of standardized protocols for early thrombosis screening. The distribution of thromboembolic event types, such as DVT, PTE, and CVT, could not be thoroughly assessed in our study due to the limited number of cases.

Nabi et al. [21] focused exclusively on patients diagnosed with glioblastoma (*N* = 1002). Of these, 162 (16.2%) developed VTE, who experienced higher rates of hospitalization, prolonged inpatient stays, and mortality (OR 4.53, 95% CI 3.01–6.80). This population consisted of older adults, with a mean age of 61.8 years, and the risk of developing DVT/PTE increased by ~ 3% for each additional year of life. Most thromboembolic events were due to PTE (56%), followed by DVT (38%) or both (6%). In another cohort, Mandel et al. [20] conducted a retrospective analysis of 282 patients with gliomas, including 49 (17.4%) with IDH-mutant and 233 (82.6%) with IDH-wild-type tumors. In total, 52 (18.4%) patients developed VTE, with a mean time to diagnosis of 2.71 months. There was no statistically significant difference in the VTE incidence between groups (*p* = 0.41). In addition, ~ 50% of the patients had isolated DVT, 19.2% had PTE, and 30.8% had both. Notably, 52% of VTE cases occurred within the first 3 months following cancer diagnosis. The mean age of patients with IDH-mutant astrocytomas was 38 years, whereas that of patients with glioblastoma was 57 years. Although both studies included tumors with varying IDH status, Mandel et al. [20] found no statistically significant difference in the VTE incidence between IDH-mutant and IDH-wild-type groups. In contrast, in our sample, the majority of thromboembolic events occurred among patients with IDH-wild-type glioblastomas. Additionally, more than half of the thromboembolic events in Mandel et al.’s [20] cohort occurred within the first 3 months following diagnosis, while in our study, it was not possible to precisely determine the timing of VTE events due to limitations in the available data. In a study by Shi et al. [22], 73 (14.84%) patients were diagnosed with DVT, confirmed through venous Doppler ultrasound, and 3 (0.61%) patients presented with both DVT and PTE.

Streiff et al. [23] conducted a prospective cohort study with 107 patients (*n* = 55, 51.4%, women; *n* = 52, 48.6%, men) with a mean age of 57 years and all diagnosed with grade 3 or 4 glioma. The authors aimed to assess the incidence of VTE and its associated risk factors. A total of 26 (24%) patients developed symptomatic VTE, most of whom had IDH-wild-type glioblastoma. Another retrospective cohort study by Helmi et al. [10] assessed the frequency of CVT exclusively in 163 patients diagnosed with IDH-wild-type glioblastoma. Of these, 12 (7.4%) patients presented with CVT before the initiation of oncologic treatment, and among them, 41.7% were also diagnosed with DVT or PTE, highlighting the importance of thorough VTE screening in this patient population. In contrast, no cases of CVT were identified in our study. This discrepancy may reflect not only the smaller sample size but also, more importantly, differences in screening strategies: while Helmi et al. [10] used systematic neuroradiological investigation, our protocol relied on clinical manifestations to prompt targeted imaging, which may have led to underdiagnosis of asymptomatic thromboses.

### Risk factors of VTE

Various studies have provided insights into the principal risk factors contributing to the occurrence of VTE, as observed through our systematic review. Hovman et al. [19] identified an advanced age, male sex, and poor functional status as predictors of VTE; our preliminary analysis also indicated that these variables may play a significant role in risk stratification, particularly among patients with glioblastoma. However, the small sample size and the lack of data regarding the impact of adjuvant treatments limit direct comparisons in terms of prognostic factors. Eisele et al. [18] found no statistically significant difference between patients with and without VTE in terms of the Karnofsky Performance Status, the extent of resection, MGMT promoter methylation status, or bevacizumab use. Our sample did not allow for a robust comparison of these outcomes. The concentration of thromboembolic events among patients with IDH-wild-type glioblastomas observed in our preliminary analysis appears to underscore the potential relevance of the tumor subtype as a risk marker, although other clinical and therapeutic factors warrant further investigation in studies with greater statistical power. According to Nabi et al. [21], the main factors contributing to this outcome are a lower Karnofsky Performance Status (40%–70%) compared to the control group, as well as the use of bevacizumab (OR 1.79, 95% CI 1.21–2.64, *p* < 0.001).

Shi et al. [22] investigated the incidence of DVT in 492 patients diagnosed with adult-type diffuse glioma. Multivariate analysis revealed that the factors most strongly associated with this outcome are prolonged surgery (lasting more than 350 min), age over 65 years, and elevated laboratory markers (e.g., activated partial thromboplastin time and D-dimer levels). Unlike the findings of Shi et al. [22], Streiff et al. [23] did not observe statistically significant differences in D-dimer levels, thrombin activity, or the ABO blood type between patients with and without VTE. However, those who underwent biopsy had up to a 3-fold increased risk of developing VTE (*p* = 0.02), and elevated factor VIII activity was associated with a 2.1-fold higher risk. As also described by Streiff et al. [23], most of the thromboembolic events in our sample too occurred in patients with IDH-wild-type glioblastomas, suggesting that the molecular subtype and tumor grade remain central factors in risk stratification, even when other clinical or laboratory markers cannot be robustly evaluated.

### Pathophysiological mechanisms

**Fig. 5:**
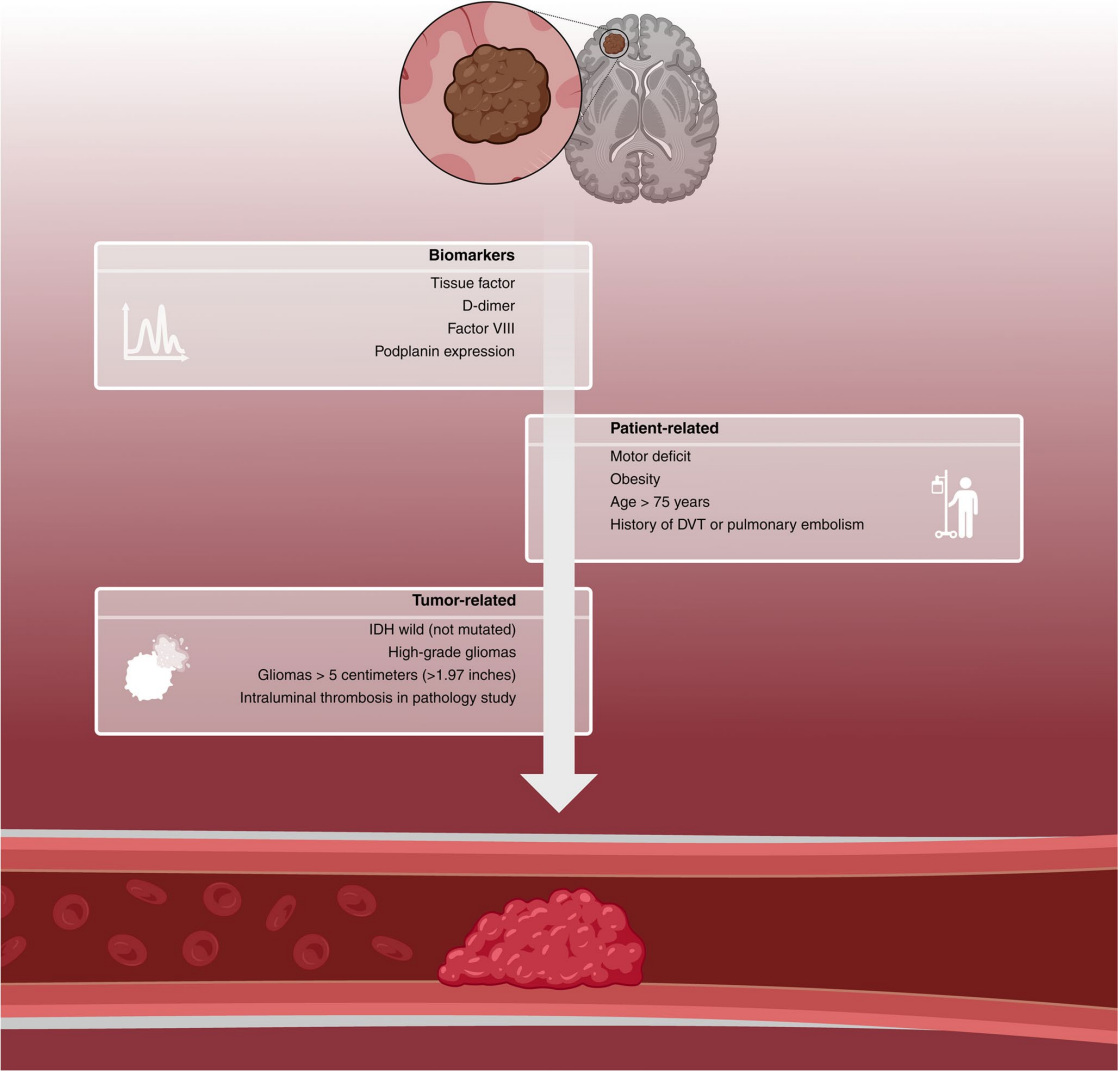
Biomarkers and patient- and tumor-related characteristics involved in the pathophysiology of VTE in patients with adult-type diffuse gliomas. Picture created on biorender.com. VTE: venous thromboembolism

**Fig. 6:**
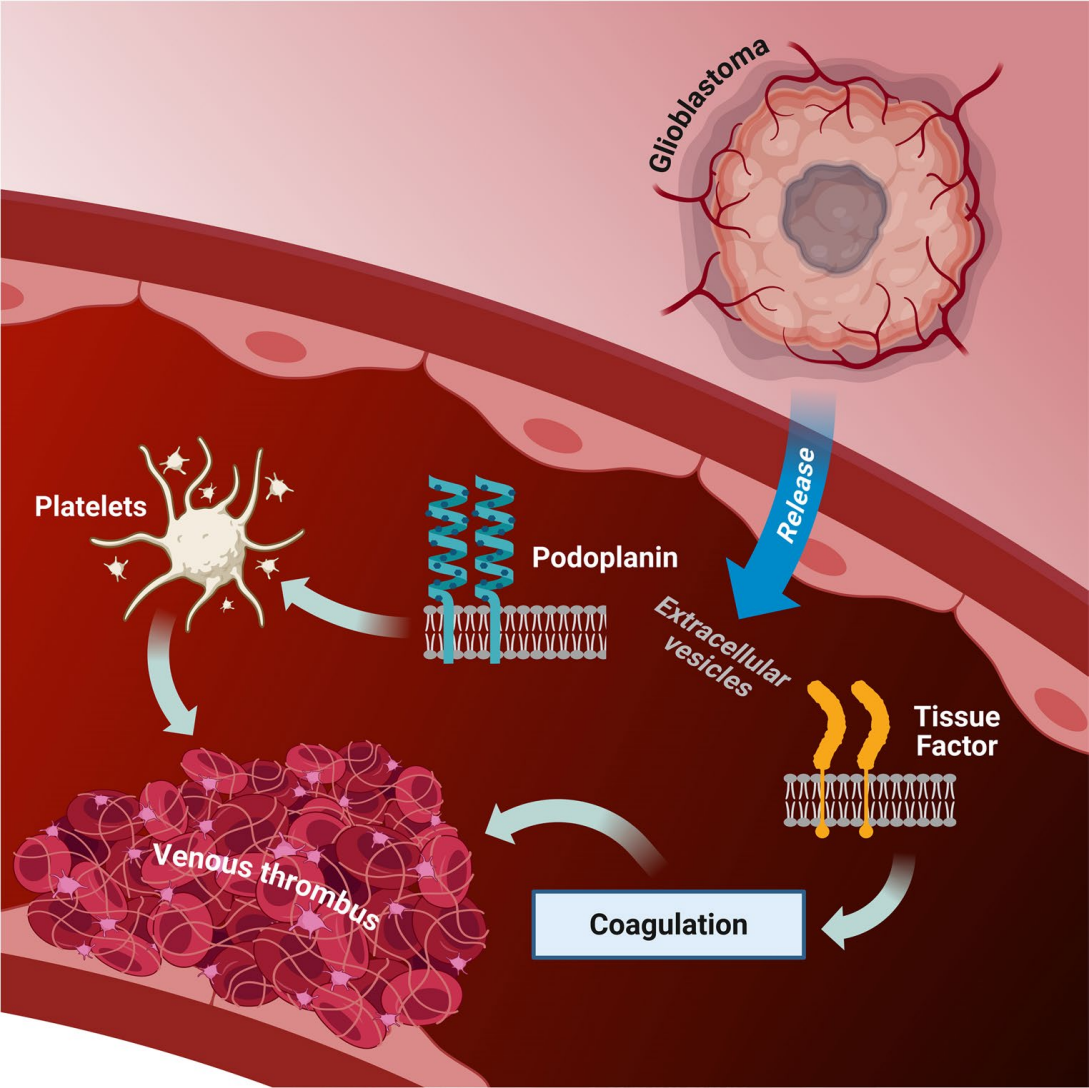
Pathways involved in VTE in patients with gliomas. Glioma cells, particularly glioblastoma cells, express podoplanin and TF and release these membrane proteins into extracellular vesicles. Podoplanin activates platelets, and TF activates the coagulation cascade, leading to venous thrombosis. Picture created on biorender.com. TF: tissue factor; VTE: venous thromboembolism

The pathophysiology of VTE in gliomas is multifactorial and complex (Figs. 5 and 6). The most frequently described mechanisms in the literature include mutations in IDH and epidermal growth factor receptor (EGFR), the expression of podoplanin and TF, and the inactivation of phosphatase and tensin homolog deleted on chromosome 10 (PTEN). Each of these mechanisms will be addressed in detail next.

#### Podoplanin

Podoplanin is a type I transmembrane glycoprotein, structurally similar to sialomucin, and is widely expressed in lymphatic endothelial cells, renal podocytes, alveolar cells, and lymph nodes, as well as brain tumors [24]. The supraphysiologic expression of podoplanin promotes increased platelet aggregation through interaction with C-type lectin-like receptor 2 (CLEC-2), ultimately favoring thrombus formation [25]. In IDH-wild-type glioblastomas, it is well established that in addition to abnormally high podoplanin expression on tumor cells, the protein is also released into the bloodstream via extracellular vesicles, contributing to reduced overall survival [26]. These patients also tend to present with elevated serum D-dimer levels and thrombocytopenia, presumably due to increased platelet consumption triggered by enhanced aggregation [25]. These procoagulant events increase the likelihood of thromboembolic complications and are associated with poorer patient outcomes [24].

#### Tissue factor

Tissue factor is a transmembrane protein expressed in various types of malignancies, initiating the coagulation cascade by binding to coagulation factors VII/VIIa [26]. It is abundantly expressed on the cell surface of high-grade gliomas [24]. After the coagulation cascade is triggered, TF leads to thrombin generation, platelet activation, and the conversion of fibrinogen into fibrin. The resulting platelet aggregation, together with fibrin formation, culminates in clot development. Additionally, through activation of protease-activated receptors, TF stimulates the secretion of pro-angiogenic factors, such as interleukin-8 and vascular endothelial growth factor (VEGF), thereby contributing to tumor progression through enhanced migration, invasion, angiogenesis, and, potentially, a hypercoagulable state [25]. Similar to podoplanin, TF is released into the bloodstream via extracellular vesicles and may act synergistically with podoplanin to amplify the thrombotic risk, as illustrated in Figs. 5 and 6 [26]. Despite the biologically plausible mechanism, clinical studies to date have not demonstrated a consistent association between TF expression in brain tumor tissue or serum levels of TF-bearing vesicles and the incidence of VTE [24].

#### IDH mutation

Mutations in IDH1 or IDH2 convert α-ketoglutarate into D-2-hydroxyglutarate, which, in turn, inhibits enzymes that require α-ketoglutarate as a cofactor, including dioxygenases involved in DNA and histone demethylation [26]. In 2016, Unruh et al. [27] demonstrated that D-2-hydroxyglutarate exerts an inhibitory effect on platelet aggregation and the coagulation cascade, and its levels are inversely correlated with TF expression. A recent meta-analysis involving 2600 patients with gliomas suggested that the presence of an IDH mutation confers a 79% lower risk of developing VTE compared to the IDH-wild-type group [28]. Another possible explanation for the reduced incidence of VTE in IDH-mutant gliomas is the lower expression of podoplanin observed in this population [24].

#### EGFR mutation

The epidermal growth factor receptor (EGFR promotes cellular differentiation and proliferation and is expressed in most human cells. It can stimulate the growth of endothelial cells by activating growth factors, such as VEGF, which, in turn, act as chemotactic factors for TF expression [29]. The *EGFR* gene is among the most frequently mutated in glioblastomas, particularly the *EGFRvIII* variant, which results from a deletion of exons 2–7. This *EGFRvIII* mutation leads to increased expression of TF, along with other procoagulant molecules, such as pseudoautosomal region (PAR)1, PAR2, and factor VII, thereby promoting a microenvironment conducive to thrombus formation [25].

#### PTEN inactivation

*PTEN* is a tumor suppressor gene involved in cell cycle regulation through inhibition of the phosphatidylinositol 3-kinase–protein kinase B (PI3K-AKT) signaling pathway. *PTEN* inactivation, which is present in up to 80% of glioblastomas, leads to overexpression of both TF and podoplanin, suggesting a role in the hypercoagulable state associated with these tumors [25]. Huang et al. [30] conducted a study involving 131 patients with gliomas in order to identify which genetic and plasma markers are most associated with thromboembolic events. The authors found that the EGFR and PTEN status was statistically significant in univariate analysis; however, these associations did not hold in multivariate analysis [30]. Thus, although there is biological plausibility for PTEN’s involvement as a procoagulant factor in gliomas, a direct correlation between PTEN status and VTE has yet to be definitively established [25].

### Limitations of our retrospective study

A retrospective cross-sectional study based on electronic medical record analysis from a single center presents several important methodological limitations:

- Selection bias: Since the study was conducted at a single institution, the results may not be generalizable to other populations or healthcare settings, limiting the sample’s representativeness.
- Data quality dependence: The accuracy and validity of findings heavily relied on the quality of electronic medical records. Incomplete, inaccurate, or inconsistently recorded data may introduce bias and compromise the reliability of the results.
- Temporal limitations: As the study was cross-sectional in design, it captured data at a single point in time, making it difficult to evaluate causality or detect temporal trends.
- Recall or documentation bias: Retrospective data are subject to potential errors or omissions in documentation, which can undermine the credibility of the collected information.
- Lack of control over variables: In retrospective studies, many relevant variables may not be consistently recorded, hindering the ability to control for confounding factors.
- Causality limitations: As the study had an observational and cross-sectional design, the findings do not allow for definitive causal inferences between the investigated variables.
- Small sample size: Our retrospective cohort was relatively small, which limits the statistical power of subgroup analyses and the generalizability of our findings. In particular, while patients with glioblastoma appeared more prone to developing VTE, this observation should be interpreted cautiously, given the limited number of thromboembolic events and potential type II errors.

### Limitations of our systematic review

Although we identified eight studies addressing VTE in patients with gliomas, the overall body of evidence remains heterogeneous and limited. There are marked differences in study design (only Streiff et al. [23] conducted a prospective follow-up; the others relied on retrospective cohorts or population databases), sample sizes (ranging from 73 to 3630 patients), WHO tumor grades included, diagnostic criteria for VTE, and follow-up durations. These variations hinder direct comparisons and preclude the possibility of summarizing incidence rates into a single, unified estimate. Furthermore, several studies were conducted at a single center and relied on hospital records, which are prone to selection bias and underreporting of thromboembolic events. In contrast, large-scale population-based analyses—although methodologically stronger—often lack critical clinical details, such as anticoagulant use, IDH mutation status, or functional performance scores. This methodological landscape—compounded by the absence of randomized trials and the lack of standardized protocols for measuring coagulation biomarkers—limits the external validity of findings and prevents robust conclusions regarding risk factors or the efficacy of primary prophylaxis. These gaps underscore the need for well-designed, multicenter studies with standardized outcome definitions and controlled methodology. In addition, we restricted our systematic literature search to studies published from 2015 onward to ensure inclusion of more contemporary cohorts, reflecting advances in diagnostics, molecular profiling, and clinical management of gliomas. However, the most recent update of the WHO classification was introduced only in 2021. Therefore, some of the included studies may not fully align with the current definitions of adult-type diffuse gliomas.

## Conclusion

Venous thromboembolism represents a clinically significant and impactful complication in patients with adult-type diffuse gliomas. In our single-center Brazilian cohort - the first from Latin America to address this topic - thromboembolic events occurred predominantly in patients with IDH–wild-type glioblastomas, particularly in the early postoperative period and among middle-aged and older adults, mirroring patterns observed in the international literature. Across the available evidence, higher tumor grade, poorer functional status, and increased surgical complexity or duration were consistently associated with increased thrombotic risk, whereas molecular features such as IDH mutation appeared to confer a protective effect. From a clinical perspective, this supports a more integrated and individualized approach to VTE risk stratification, incorporating clinical, surgical, and molecular factors to guide surveillance and prophylactic strategies. At the same time, the current evidence base remains heterogeneous and limited, with predominantly retrospective data and inconsistent reporting of key variables. Future research should therefore prioritize large, prospective, and molecularly annotated cohorts, as well as pragmatic studies aimed at optimizing thromboprophylaxis while balancing hemorrhagic risks. 

## Supplementary Information

Below is the link to the electronic supplementary material.

Supplementary Material 1

Supplementary Material 2

## Data Availability

No datasets were generated or analyzed during this study.
