# Supplementary material for: Assessment of venous thromboembolism in adult-type diffuse gliomas at a quaternary neuro-oncology center: a retrospective cross-sectional study and systematic review
Source: Neurosurg Rev. 2026 Apr 25;49(1):377. doi: 10.1007/s10143-026-04299-6 (PMC13109160; doi:10.1007/s10143-026-04299-6)
Supplement: Supplementary file 1 — Supplementary Material 1 [file 10143_2026_4299_MOESM1_ESM.docx]

**Online Resource 2**

*Neurosurgical Review*

~

**Assessment of venous thromboembolism in adult-type diffuse gliomas at a quaternary neuro-oncology center: a retrospective cross-sectional study and systematic review**

Leonardo de Sousa Bernardes^1^, Lucas de Oliveira Woehl^2^, Jean Gonçalves de Oliveira^3^, José Carlos Esteves Veiga^3^, João Luiz Vitorino Araujo^3^

^1^ Department of Neurology, Santa Casa de São Paulo School of Medical Sciences, São Paulo, SP, Brazil

^2^ Universidade do Planalto Catarinense, Lages, SC, Brazil

^3^ Division of Neurosurgery, Santa Casa de São Paulo School of Medical Sciences, São Paulo, SP, Brazil

**Corresponding author:**

Leonardo de Sousa Bernardes, MD,

Department of Neurology, Santa Casa de São Paulo, São Paulo, SP, Brazil.

Email: [leonardo.ds.bernardes@gmail.com](mailto:leonardo.ds.bernardes@gmail.com)

*Supplementary Table 2*. Reason for the exclusion of assessed reports.

| Author/year | Link | Reason for exclusion |
| --- | --- | --- |
| Abdelmessih et al 2023 | <https://ashpublications.org/blood/article/142/Supplement%201/5122/505901/Tolerability-of-Direct-Oral-Anticoagulants-and-Low> | Population |
| Becattini et al 2023 | <https://pubmed.ncbi.nlm.nih.gov/37810416/> | Population |
| Bianconi et al 2021 | <https://pubmed.ncbi.nlm.nih.gov/34763392/> | Design |
| Burth et al 2021 | <https://pubmed.ncbi.nlm.nih.gov/33674992/> | Outcomes |
| Carney et al 2018 | <https://ashpublications.org/blood/article/132/Supplement%201/2521/263991/Safety-of-Direct-Acting-Oral-Anticoagulants-Versus> | Other* |
| Carney et al 2019 | <https://ashpublications.org/blood/article/134/Supplement_1/2438/422949/Recurrent-Intracranial-Hemorrhage-and-Venous> | Other* |
| Chai-Adisaksopha et al 2017 | <https://pubmed.ncbi.nlm.nih.gov/28078351/> | Population |
| Costa et al 2015 | <https://ascopubs.org/doi/10.1200/jco.2015.33.15_suppl.e13059> | Design |
| Cote et al 2016 | <https://pubmed.ncbi.nlm.nih.gov/27764877/> | Design |
| Craven et al 2018 | <https://pubmed.ncbi.nlm.nih.gov/29693482/> | Population |
| Czap et al 2019 | <https://pubmed.ncbi.nlm.nih.gov/31041798/> | Design |
| Diaz and Jo 2022 | <https://pubmed.ncbi.nlm.nih.gov/35179708/> | Design |
| Diaz and Schiff 2022 | <https://pubmed.ncbi.nlm.nih.gov/35788556/> | Design |
| Diaz et al 2020 | <https://pubmed.ncbi.nlm.nih.gov/33361259/> | Outcomes |
| Donoho et al 2018 | <https://pubmed.ncbi.nlm.nih.gov/28988350/> | Outcomes |
| Dubinski et al 2022 | <https://pubmed.ncbi.nlm.nih.gov/33900495/> | Outcomes |
| Edwin et al 2015 | <https://ascopubs.org/doi/10.1200/jco.2015.33.15_suppl.e13027> | Other* |
| Gok et al 2023 | <https://pubmed.ncbi.nlm.nih.gov/36066053/> | Population |
| Gonzalez-Delgado et al 2023 | <https://pubmed.ncbi.nlm.nih.gov/37178770/> | Design |
| Gramatzki et al 2020 | <https://ascopubs.org/doi/10.1200/JCO.2020.38.15_suppl.e14530> | Other* |
| Hassan et al 2023 | <https://ascopubs.org/doi/10.1200/JCO.2023.41.16_suppl.e14044> | Other* |
| Heenkenda et al 2019 | <https://pubmed.ncbi.nlm.nih.gov/31677594/> | Outcomes |
| Helmi et al 2019 | <https://pubmed.ncbi.nlm.nih.gov/30684707/> | Outcomes |
| Jeraq et al 2017 | <https://pubmed.ncbi.nlm.nih.gov/27628002/> | Design |
| Jo et al 2020 | <https://academic.oup.com/neuro-oncology/article/20/suppl_6/vi200/5154162> | Other* |
| Jo et al 2023 | <https://pubmed.ncbi.nlm.nih.gov/37100086/> | Design |
| Kabashneh et al | <https://pubmed.ncbi.nlm.nih.gov/32714708/> | Design |
| Kapteijn et al 2021 | <https://academic.oup.com/neuro-oncology/article/23/Supplement_2/ii20/6366789> | Other* |
| Kapteijn et al 2024 | <https://pubmed.ncbi.nlm.nih.gov/39168144/> | Design |
| Lomax and Kong et al 2023 | <https://academic.oup.com/neuro-oncology/article/25/Supplement_5/v217/7406530> | Other* |
| Majarafi et al 2024 | <https://ascopubs.org/doi/10.1200/JCO.2024.42.16_suppl.e14040> | Other* |
| Mandel et al 2020 | <https://www.neurology.org/doi/10.1212/WNL.94.15_supplement.2651> | Other* |
| Mantia et al 2017 | <https://pubmed.ncbi.nlm.nih.gov/28468796/> | Population |
| Martín et al 2014 | <https://pubmed.ncbi.nlm.nih.gov/24643701> | Population |
| Muster and Gary et al 2020 | <https://pubmed.ncbi.nlm.nih.gov/32466430/> | Design |
| Muster and Gary et al 2021 | <https://pubmed.ncbi.nlm.nih.gov/34200229/> | Design |
| Okamoto et al 2022 | <https://pubmed.ncbi.nlm.nih.gov/35173104/> | Population |
| Orešković et al 2021 | <https://pubmed.ncbi.nlm.nih.gov/34717073/> | Design |
| Poon et al 2023 | <https://pubmed.ncbi.nlm.nih.gov/37953241/> | Population |
| Qian et al 2016 | <https://pubmed.ncbi.nlm.nih.gov/26610747/> | Design |
| Rahmani et al 2020 | <https://pubmed.ncbi.nlm.nih.gov/31704036/> | Population |
| Ranjan et al 2024 | <https://pubmed.ncbi.nlm.nih.gov/38288941/> | Design |
| Rhun et al 2018 | <https://pubmed.ncbi.nlm.nih.gov/30036741> | Outcomes |
| Riedl and Ay 2019 | <https://pubmed.ncbi.nlm.nih.gov/31041803/> | Design |
| Sloan et al 2022 | <https://pmc.ncbi.nlm.nih.gov/articles/PMC9661167/> | Other* |
| Smith et al 2015 | <https://pubmed.ncbi.nlm.nih.gov/25533212/> | Population |
| Supbumrung et al 2023 | <https://pubmed.ncbi.nlm.nih.gov/38039526/> | Design |
| Unruh et al 2016 | <https://pubmed.ncbi.nlm.nih.gov/27664011/> | Design |
| Veiga et al 2024 | <https://pmc.ncbi.nlm.nih.gov/articles/PMC11189257/> | Design |
| Wilhelmy et al 2023 | <https://pubmed.ncbi.nlm.nih.gov/38066037/> | Outcomes |
| Woods and Lesser 2023 | <https://pubmed.ncbi.nlm.nih.gov/37407888/> | Design |
| Yang et al 2023 | <https://pubmed.ncbi.nlm.nih.gov/37413715/> | Design |
| Yust-Katz et al 2019 | <https://academic.oup.com/neuro-oncology/article/21/Supplement_6/vi204/5619537> | Other* |
| Zeng et al 2024 | <https://pubmed.ncbi.nlm.nih.gov/37947983/> | Design |
| Zhang et al 2021 | [https://pubmed.ncbi.nlm.nih.gov/33581794/](http://ncbi.nlm.nih.gov/33581794/) | Population |
| Zhang et al 2023 | <https://pubmed.ncbi.nlm.nih.gov/36805800/> | Other* |
| Zwicker et al 2016 | <https://pubmed.ncbi.nlm.nih.gov/27306689/> | Design |

*Other: preclinical studies, abstracts, or preprints.
