# Supplementary material for: Assessment of venous thromboembolism in adult-type diffuse gliomas at a quaternary neuro-oncology center: a retrospective cross-sectional study and systematic review
Source: Neurosurg Rev. 2026 Apr 25;49(1):377. doi: 10.1007/s10143-026-04299-6 (PMC13109160; doi:10.1007/s10143-026-04299-6)
Supplement: Supplementary file 2 — Supplementary Material 2 [file 10143_2026_4299_MOESM2_ESM.docx]

**Online Resource 1**

*Neurosurgical Review*

~

**Assessment of venous thromboembolism in adult-type diffuse gliomas at a quaternary neuro-oncology center: a retrospective cross-sectional study and systematic review**

Leonardo de Sousa Bernardes^1^, Lucas de Oliveira Woehl^2^, Jean Gonçalves de Oliveira^3^, José Carlos Esteves Veiga^3^, João Luiz Vitorino Araujo^3^

^1^ Department of Neurology, Santa Casa de São Paulo School of Medical Sciences, São Paulo, SP, Brazil

^2^ Universidade do Planalto Catarinense, Lages, SC, Brazil

^3^ Division of Neurosurgery, Santa Casa de São Paulo School of Medical Sciences, São Paulo, SP, Brazil

**Corresponding author:**

Leonardo de Sousa Bernardes, MD,

Deprtament of Neurology, Santa Casa de São Paulo, São Paulo, SP, Brazil.

Email: [leonardo.ds.bernardes@gmail.com](mailto:leonardo.ds.bernardes@gmail.com)

*Supplementary Table 1*. Search strategy based on electronic databases.

| Database | PubMed | |
| --- | --- | --- |
| #1 | ("Glioma"[Mesh] OR "Glioblastoma"[Mesh] OR glioma OR "glioblastoma multiforme" OR GBM OR "high-grade glioma" OR astrocytoma OR "brain tumor" OR "brain neoplasm" OR "CNS tumor" OR "central nervous system tumor") | |
| #2 | ("Venous Thromboembolism"[Mesh] OR VTE OR "venous thromboembolism" OR "deep vein thrombosis" OR DVT OR "pulmonary embolism" OR PE OR "thrombosis" OR "venous thrombosis" OR "blood clot" OR "thromboembolic event") | |
| #3 | ("prevalence" OR "disease prevalence" OR "point prevalence" OR "incidence" OR "disease incidence" OR "risk factors" OR determinants OR predictors OR "epidemiology" OR "disease burden" OR "patterns of disease" OR "frequency") | |
| #4 | ("cross-sectional studies"[Mesh] OR "cohort studies"[Mesh] OR "longitudinal studies" OR "case-control studies"[Mesh] OR "population-based studies" OR "registry-based studies" OR "descriptive studies") | |
| #5 | ("2015/01/01"[Date - Publication] : "2024"[Date - Publication]) | |
| #6 | "humans"[MeSH Terms] | |
| #7 | #1 AND #2 AND #3 AND #4 AND #5 AND #6 AND #7 | |
| Number of results | 108 | |
| Database | EMBASE (Elsevier) | |
| #1 | ('glioma'/exp OR 'glioblastoma'/exp OR glioma OR 'glioblastoma multiforme' OR gbm OR 'high-grade glioma' OR astrocytoma OR 'brain tumor' OR 'brain neoplasm' OR 'cns tumor' OR 'central nervous system tumor') | |
| #2 | ('venous thromboembolism'/exp OR vte OR 'venous thromboembolism' OR 'deep vein thrombosis' OR dvt OR 'pulmonary embolism' OR pe OR 'thrombosis' OR 'venous thrombosis' OR 'blood clot' OR 'thromboembolic event') | |
| #3 | ('prevalence'/exp OR 'disease prevalence' OR 'point prevalence' OR 'incidence'/exp OR 'disease incidence' OR 'risk factors'/exp OR determinants OR predictors OR 'epidemiology'/exp OR 'disease burden' OR 'patterns of disease' OR 'frequency') | |
| #4 | ('cross-sectional study'/exp OR 'cohort study'/exp OR 'longitudinal study' OR 'case-control study'/exp OR 'population-based study' OR 'registry-based study' OR 'descriptive study') | |
| #5 | ([2015-2024]/py) | |
| #6 | 'human'/de | |
| #7 | #1 AND #2 AND #3 AND #4 AND #5 AND #6 AND #7 | |
| Number of results | 279 | |
| Database | SCOPUS (Elsevier) | |
| #1 | TITLE-ABS-KEY ("glioma" OR "glioblastoma" OR "glioblastoma multiforme" OR "GBM" OR "high-grade glioma" OR "astrocytoma" OR "brain tumor" OR "brain neoplasm" OR "CNS tumor" OR "central nervous system tumor") | |
| #2 | TITLE-ABS-KEY ("venous thromboembolism" OR "VTE" OR "deep vein thrombosis" OR "DVT" OR "pulmonary embolism" OR "PE" OR "thrombosis" OR "venous thrombosis" OR "blood clot" OR "thromboembolic event") | |
| #3 | TITLE-ABS-KEY ("prevalence" OR "disease prevalence" OR "point prevalence" OR "incidence" OR "disease incidence" OR "risk factors" OR "determinants" OR "predictors" OR "epidemiology" OR "disease burden" OR "patterns of disease" OR "frequency") | |
| #4 | TITLE-ABS-KEY ("cross-sectional studies" OR "cohort studies" OR "longitudinal studies" OR "case-control studies" OR "population-based studies" OR "registry-based studies" OR "descriptive studies") | |
| #5 | #1 AND #2 AND #3 AND #4 | |
| #6 | #5 with filter: custom range 2015 to 2024 | |
| Number of results | 108 | |
| Database | Web of Science | |
| #1 | ("glioma" OR "glioblastoma" OR "glioblastoma multiforme" OR "GBM" OR "high-grade glioma" OR "astrocytoma" OR "brain tumor" OR "brain neoplasm" OR "CNS tumor" OR "central nervous system tumor") | |
| #2 | ("venous thromboembolism" OR "VTE" OR "deep vein thrombosis" OR "DVT" OR "pulmonary embolism" OR "PE" OR "thrombosis" OR "blood clot" OR "thromboembolic event") | |
| #3 | ("prevalence" OR "incidence" OR "risk factors" OR "epidemiology" OR "disease burden" OR "patterns of disease" OR "frequency") | |
| #4 | PY=(2015-2024) | |
| #5 | #1 AND #2 AND #3 AND #4 | |
| Number of results | 200 | |
